# Rhenium-188 Production in Hospitals, by W-188/Re-188 Generator, for Easy Use in Radionuclide Therapy

**DOI:** 10.1155/2013/290750

**Published:** 2013-04-09

**Authors:** Maria Argyrou, Alexia Valassi, Maria Andreou, Maria Lyra

**Affiliations:** ^1^1st Radiology Department, National & Kapodistrian University of Athens, Athens, Greece; ^2^Medical Physics Laboratory, School of Medicine, National & Kapodistrian University of Athens, Athens, Greece; ^3^Radiation Physics Unit & Division of Nuclear Medicine, 1st Radiology Department, National & Kapodistrian University of Athens, Athens, Greece

## Abstract

Rhenium-188 (Re-188) is a high energy *β*-emitting radioisotope obtained from the tungsten-188/rhenium-188 (W-188/Re-188) generator, which has shown utility for a variety of therapeutic applications in nuclear medicine, oncology, and interventional radiology/cardiology. Re-188 decay is accompanied by a 155 keV predominant energy *γ*-emission, which could be detected by *γ*-cameras, for imaging, biodistribution, or absorbed radiation dose studies. Its attractive physical properties and its potential low cost associated with a long-lived parent make it an interesting option for clinical use. The setup and daily use of W-188/Re-188 generator in hospital nuclear medicine departments are discussed in detail. The clinical efficacy, for several therapeutic applications, of a variety of Re-188-labeled agents is demonstrated. The high energy of the *β*-emission of Re-188 is particularly well suited for effective penetration in solid tumours. Its total radiation dose delivered to tissues is comparable to other radionuclides used in therapy. Furthermore, radiation safety and shielding requirements are an important subject of matter. In the case of bone metastases treatment, therapeutic ratios are presented in order to describe the efficacy of Re-188 usage.

## 1. The W-188/Re-188 Generator

### 1.1. W-188 Production

Re-188 is carrier free as Na^188^ *Re*O_4_ obtained from a W-188/Re-188 alumina based generator. Parent radionuclide, tungsten-188 (W-188) with a half life equal to 69.4 d, is produced in a nuclear reactor by irradiation of tungsten oxide, 96.07% enrichment in tungsten-188 (W-188) with thermal and high energy neutrons. The decay chain from a 100% pure target W-186 [1] according to neutron flux and irradiation time shows the origin of some of the radioactive contaminants present in the generators. Iridium-192 and osmium-191 are formed during reactor irradiation of tungsten-186 and are thus always present in reactor-produced tungsten-188.These impurities can be removed while obtaining Re-188, during the generator elution process and subsequent postelution passage through alumina. Because the production of tungsten-188 from enriched tungsten-186 is a double neutron capture process, the production yield is a function of the square of the neutron flux. For this reason, a very high neutron flux greater than 8–10 × 10^14^ n/cm^2^/s is required for the production of tungsten-188. There are five reactors in the world (the High Flux Isotope Reactor in Oak Ridge National Laboratory, the High Flux Beam Reactor in Brookhaven National Laboratory, the Missouri University Research Reactor, the Fast Flux Test Facility in Westinghouse Hanford and the Japan Materials Testing Reactor), providing such high neutron fluxes and irradiation times varying to 24 hours and 60 days (see [2, Table 1] and [3]). The processing of tungsten-188 is generally conducted in a quartz glass vessel. Processing of the granular tungsten metal and tungsten oxide targets involves dissolution in sodium hydroxide solution containing hydrogen peroxide solution to form sodium tungstate. The solution is then filtered and a germanium detector is used in order to determine tungsten-188, Os-191, and Ir-192 levels and any other impurity which may be present. Processed tungsten-188 is generally stored as sodium tungstate in sodium hydroxide solution, which is acidified just prior to loading. For large, clinical-scale generators (500–1000 mCi tungsten-188), it is convenient to have specific volume solutions greater than 20 mCi/mL.

### 1.2. Re-188 Production

**Figure 1:**
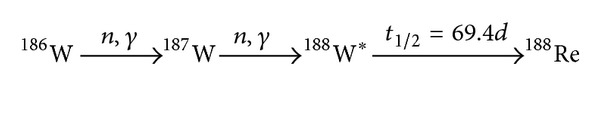
Decay scheme for Re-188 production.

Rhenium-188 is produced according to the decay scheme in Figure 1 and its main physical properties are shown in Table 1. Concentration of the Re-188 elutant is of great importance and quality control of preparation of Re-188 radiopharmaceuticals is significant for proper biodistribution and therapeatic results. Two 55 GBq generators can provide sufficient activity for therapeutic administrations of Re-188 radiopharmaceuticals during a period of one year. Setup and use of the W-188/Re-188 generator and handling of high-level Re-188 solutions require adequate attention to the radiation safety. The transarterial administration of Re-188-labeled agents for treatment of inoperable liver cancer requires use of high-level generators. The handling of such high levels of Re-188 imposes radiological precautions and adequate care that covers the ALARA principles (as low as reasonably achievable). 

**Figure 2:**
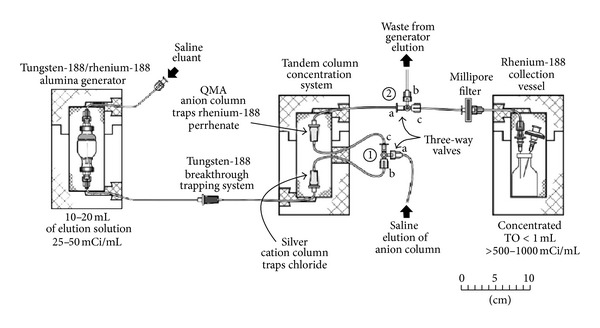
Typical W-188/Re-188 generator setup [5].

A W-188/Re-188 generator similar to the prototype in the Oak Ridge National Laboratory (ORNL) [5] is based on the use of an aluminum oxide column permitting elution with normal saline. The use of this generator system in various institutions has demonstrated high yields of Re-188 accompanied with low W-188 parent breakthrough during periods of several months. The alumina-based W-188/Re-188 generators are produced by absorption of reactor-produced W-188 as tungstic acid on a column of alumina, with a typical maximum loading of 50 mg W per gram of alumina. The generators are washed slowly (1 mL/min) with saline (150–200 mL) after W-188 loading and air dried before shipment. Generators are provided with short lengths of arterial extension tubing that are coiled into recessed holes at the top inlet and bottom outlet of the lead shielding unit. To minimize exposure to personnel, after receipt and unpacking, the system is typically housed behind a leaded glass and/or polyacrylate shield. The typical generator system configuration is shown in Figure 2. In order to minimize the radiation exposure caused by the bremsstrahlung from interaction of beta particles with the lead, a polyacrylate shield between the generator and solutions of Re-188 and the lead shield is used. An in-line acidic alumina trapping column effectively removes the low levels of any W-188 parent breakthrough and any alumina or other particles which may be eluted from the generator. 

**Figure 3:**
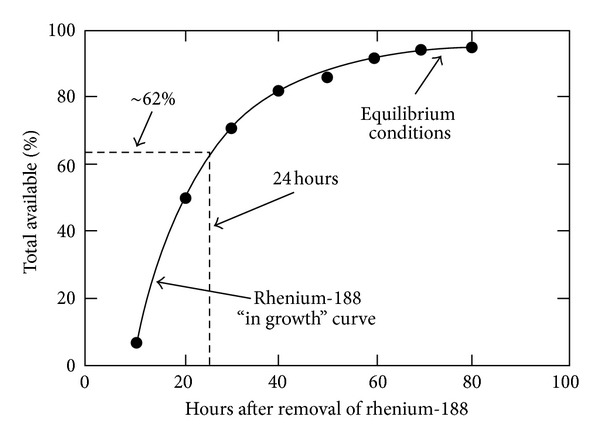
Elution yields of Re-188 in the W-188/Re-188 generator [5].

The Re-188 yields after elution are generally 75% to 80% of the available Re-188. The generator reaches the 62% of the equilibrium after 24 hours; therefore, the daily elution will provide approximately 50% of the Re-188 that would be available at equilibrium, adjusting the availability of Re-188 for the preparation of therapeutic agents in a daily basis. The breakthrough values of W-188 are usually very small (in the 10^−6^ range). In other case, any break through can be effectively removed by subsequent postelution passage of the bolus through the alumina column. A typical large clinical-scale generator loaded with greater than 1 Ci of W-188 provides more than 750 mCi (greater than 75% yield) of Re-188 perrhenate at equilibrium (30–35 mCi/mL) or approximately 500 mCi (20–25 mCi/mL) for sequential daily elutions (Figure 3). The high Re-188 yields and low W-188 breakthrough can be maintained during at least 60 days with the alumina-based generator. The high activity levels (1-2 Ci) of Re-188 solution require special treatment of the radiological protection issues. In addition to the bremsstrahlung radiation mentioned above, there are high energy gamma rays emitted by Re-188 and thus extra shielding for gamma camera imaging is required. For this purpose, medium or high energy collimation should be used instead of the conventional type of collimation for the 155 keV photons. Nevertheless, careful monitoring of radiation levels and especially use of ring monitors for extremities exposure measurements are required. Furthermore, personnel exposures may be reduced by automation or semiautomation of generator operation. Rhenium radiopharmaceuticals are prepared from the permetallate ions obtained from generators. The metal ions must be reduced by an appropriate reducing agent and coordinated by ligand systems, which stabilize the lower oxidation states of the metals and significantly determine the biological distribution patterns of the pharmaceuticals. In this stage, the use of free radical scavengers (e.g., ethanol of 2%) is suggested in order to avoid decomposition of the radiochemical solution due to the ionizing radiation action. In this way, a much longer shelf life is achievable. 

High activity-concentration Re 188 is necessary for the preparation of some Re 188-labeled pharmaceuticals. Several methods based on generator postelution concentration of Re-188 elutant have been developed aiming to increase the activity concentration. The alumina trapping column is a safety measure that is highly recommended for use with such type of generators. The low tungsten/alumina loading capacity (less than 50 mg W/gm alumina) prevents increased W breakthrough during long-term usage. However, the elution volumes are much larger than those for high-capacity generators. This could result in an increased backpressure during positive elution. After anion column trapping and subsequent elution with saline, very high purity solutions of Re-188 are obtained. 

 The long useful life of the W-188/Re-188 generator is a major reason for the cost-effectiveness of Re-188 radiopharmaceuticals. Costs of transport of the generator and of consumable supplies are modest even if column concentration of eluates is necessary for Re-188 radiopharmaceutical preparation.

## 2. Clinical Applications

 Rhenium-188 is widely used in clinical applications because of important advantages over other radioisotopes. First of all, it is available onsite from the tungsten-188/Re-188 generator in the majority of hospitals, in a convenient and inexpensive way. It has a physical half life which is appropriate for therapeutic purposes, reducing the toxicity risks, compared to other radioisotopes (*t*_1/2_ = 1.95 d for Sm-153, *t*_1/2_ = 53 d for Sr-89). It is chemically similar to technetium and many biological results already obtained for the latter could be exploited. However, the reduction potential for Re is substantially larger and therefore a much greater quantity of reducing agent is required. Given that its chemical reactions are mostly redox, Re-188 resolves as anion *Re*O_4_^−1^, resulting in rapid secretion from vital organs. The penetration depth is larger in relation to Sr-89 and Sm-153. The energy of *γ*-radiation emitted is detectable by *γ*-cameras, for imaging, biodistribution, or absorbed radiation dose studies whereas no extra shielding is required because it does not differ substantially from the energy emitted by ^99m^Tc decay.

### 2.1. Chemical Forms of Re-188

 Because of its physical properties, Re-188 is suitable for labeling peptides, antibodies, and colloids to form radiopharmaceuticals. The most common forms of Re-188 radiopharmaceuticals are [6] described below. Re-188 HEDP (hydroxyethylidenediphosphonate)/DMSA (dimercaptosuccinic acid) is used in osseous metastases which stem from prostate and breast cancer. In most studies, a dose 30 mCi–90 mCi of Re-188 HEDP is injected intravenously and whole-body dynamic scans are obtained 1 to 6 days later. Re-188/HEDP is mostly concentrated on bone metastases and its excretion rate through urine is 62% of the administered activity within the first 2 days [7]. The biodistribution of Re-188/DMSA is similar to ^99m^Tc analogue. Re-188 DMSA shows selectivity for bone metastases and kidney, but uptake in normal bone is not significantly greater than in surrounding soft tissues [8]. Re-188 HDD (Lipiodol) (4-hexadecyl-1, 2, 9, 9-tetramethyl-4, 7-diaza-1,10-decanethiol) is also utilized for the treatment of hepatocellular carcinomas (HCC) [5]. The administration of the radiopharmaceutical is happening directly to the liver through a catheter which is inserted transfemorally and introduced into the proper hepatic artery [9]. The injected activity is cleared fast in the blood with an effective half life of 7.6 ± 2.2 h. The predominant elimination of the activity is observed through urinary excretion with a mean renal clearance of 44.1 ± 11.7% of the injected activity within the 76 h after administration. The calculated whole-body effective half life is 14.3 ± 0.9 h. Other Re-188 labeled radiopharmaceuticals were developed for liver cancer treatment. Lambert et al. [9] have performed a comparative study concerning the most widely used radiopharmaceuticals for this purpose. The dose range in liver has been found to lie in the range 4.5–21.8 Gy if the administered activity is 3.7−7 GBq [10]. The use of Re-188 HDD suggests a high dose tolerance in patients with Child-Pugh A cirrhosis. However, the labeled yield is rather small (50%–70%) and thus the possibility to achieve high therapeutic activity is limited. For that reason a number of studies [11–15] have focused on the development of new radiopharmaceuticals with high yield. These include Re-188 N-DEDC, Re-188-SSS Lipiodol, and Re-188 human serum albumin or HSA B20 microspheres (range  15–37 m). The labeling yields are 95% for Re-188 N-DEDC, 97% for Re-188-SSS Lipiodol and 80% for human serum albumins (HSA) microspheres as a microembolising vehicle for Re-188. The advantages of Re-188 radiopharmaceuticals over those of I-131 were also highlighted. The lower energy emitted, half life, the on-site availability, and nontoxicity prevent radiation protection issues. Comparing Re-188 to Y-90 microspheres, the treatment with Re-188 Lipiodol is a more simple process and can be performed to all referred patients with hepatocellular carcinoma (HCC). The drop-out risk is reduced, and no distortion or destruction of the arterial supply is observed; therefore, multiple treatment sessions could easily be performed. R-188-anti-CD20 is used for the treatment of non-Hodgkin's lymphoma. According to Garcia et al. [16], thirty minutes after administration the percentage of the injected activity in the liver, spleen, and kidneys was 22.5% ± 5.2%, 4.5% ± 2.1% and 2.1% ± 0.6%, respectively. After 24 h, the liver, spleen, and kidneys activity decreased to, 5.5% ± 0.4%, 1.4% ± 0.2% and 0.52 % ±  0.10% of injected activity, respectively.

Skeletal metastases are the most common form of metastatic cancer, appearing in the vast majority of patients with breast and prostate cancer and frequently in patients with lung cancer, renal cancer, thyroid cancer, and multiple myeloma. These metastases are a major cause of serious morbidity resulting in severe bone pain, hypocalcaemia, and loss of function following pathological fractures and neurological symptoms from nerve compression. 

Radionuclide therapy of bone metastases was first used decades ago by administration of P-32. Phosphorus [17] is incorporated in the DNA of rapidly increasing bone marrow cell and in structures of trabecular and cortical bone. For therapy purposes, radioisotopes similar either to calcium or to phosphonate carrier molecules are used. The former category includes Sr-89 chloride or Y-90. These are accumulated as cations in the bone and in relation to the osseous metabolism. In the latter category, Sm-153 EDTMP and Re-186 HEDP are known to have an excellent radio affinity. Re-188 is more attractive than Re-186 because it can be obtained in noncarrier form from a W-188/Re-188 generator and has a long shelf life of several months [18]. Re-188 HEDP palliative treatment is recommended for patients with persisting bone pains after chemotherapy or external beam therapy and having life expectancy at least 6 months (corresponding to Karnofsky index over 30%). Pregnant patients under 18 years old subjected to chemotherapy or having renal failures should be excluded. Treatment is usually performed in accordance with the detailed guidelines of the European Association of Nuclear Medicine regarding radionuclide administration for palliative purposes [19].

### 2.2. Dosimetry of Re-188 HEDP in Skeletal Metastases

The assessment of the absorbed dose to organs tissues is a critical step in patient treatment planning within radioimmunotherapy. Two generalized approaches have been applied to patient organ dosimetry in therapeutic nuclear medicine. The more straightforward approach is to estimate the absorbed dose to organs of interest using anatomic phantoms representative of each patient or, at least, an average patient. Related techniques are currently implemented in the OLINDA/EXM software [20] using the stylized phantom series of the Oak Ridge National Laboratory (ORNL) and methods outlined in [21]. The main advantage of this method is its ease of use, but only the average absorbed dose can be estimated. Neither heterogeneities in either tissue composition or radioactivity distribution nor the anatomic geometry of the source and target tissues within the body of the patient is considered. As an alternative approach, voxelized geometries may be used to represent the organs and tumors as tagged voxels of different activity levels, and Monte Carlo (MC) radiation transport can be used to assess dose at the voxel level. The average absorbed dose may be obtained by tracking energy deposition events within the target organ and assembling a dose-volume histogram (DVH) through computation of the absorbed dose at each voxel within the organ. The advantage of this technique is that it can handle tissue heterogeneities (bone, soft tissue, air, lung, etc.) and take into account the patient-specific anatomic geometry of all source and target tissues. However, this approach is time consuming and computationally demanding. For a given organ of interest, tissue heterogeneities are not generally present. Thus, characterization of dose per organ voxel can be conveniently handled, as described in [22], via an image-based map of time-integrated activity in each voxel of the organ, and radionuclide S values developed at the voxel level.

The efficacy of Re-188 HEDP therapy in bone pain palliation was evaluated by Liepe et al. [23, 24] in a group of 27 patients with hormone refractory prostate carcinoma. The patients were treated with doses of 2.7–3.4 GBq of Re-188 and they showed a response rate of 76% and reduction of analgesic intake and pain intensity. Dosimetric calculations were based on MIRD schema and SPECT data. The volume of bone metastases was calculated by posttherapeutic SPECT data after number-of-voxels determination. Determination of radiation-absorbed doses requires to take into consideration the complexity of osseous tissue consisting of cortical and trabecular structures as well as bone marrow. According to MIRD schema [25], the absorbed dose is given by

(1) $$\begin{array}{c} {\tilde{D}}_{k}=A_{0}\tau_{j}\;\sum_{j}\;S_{j}\left(r_{j}\to r_{k}\right), \end{array}$$

where *S*_*j*_(*r*_*j*_ → *r*_*k*_) is the *S* value, the mean absorbed dose per unit accumulated activity with *j* as the source and *k* as the target, and *τ*_*j*_ is the residence time, that is, the ratio of the cumulated activity ($\tilde{A}$) and the administered activity (*A*_0_). The cumulated activity is the time integral of activity in a specific ROI,

(2) $$\begin{array}{c} \tilde{A}={\int_{0}}^{\infty }A\left(t\right)dt. \end{array}$$

The distribution of the radionuclide in the metastases was assumed to be homogeneous. The volume and residence time data were entered into the nodule module option of the MIRDOSE 3.1 program [26] to obtain the specific radiation-absorbed dose values for assumed soft-tissue tumors. 

The mean specific radiation-absorbed dose in bone metastases according to Liepe et al. [24] (3.83 ± 2.01 mGy/MBq) was found to be 67 times higher than the dose in bone marrow (0.61 ± 0.21 mGy/MBq). Because of the rapid urinary excretion rate, the radiation absorbed dose to the whole body was low (0.07 ± 0.02 mGy/MBq). Furthermore, a mean dose value of 0.6 ± 0.2 mGy/MBq for the red bone marrow did not lead to any clinically significant thrombocytopenia or leukopenia. In another study conducted by Savio et al. [27], 21 patients received 1.3 or 2.2 GBq, in single or multiple doses. Absorbed dose in bone marrow was estimated with MIRDOSE3. Single doses of low activity (1.3 GBq) were given to 12 patients. Nine patients received multiple doses. The dosimetric estimations for absorbed doses after single or multipl Re-188-HEDP administration were (2.3 ± 0.9) cGy/37 MBq (1 mCi) bone marrow dose (BMD) and (71 ± 37) cGy total body marrow dose (TBMD) for the first group of patients (received single dose), while they are (1.6 ± 0.9) cGy/37 MBq (1 mCi) BMD and (83 ± 55) cGy TBMD for the second group (received multiple doses). 

 Maxon et al. [28] evaluate the Re-188-(Sn)HEDP as a radiopharmaceutical that localizes in skeletal metastases based on the prior experience of Re-188-HEDP. In vivo and in vitro tests were conducted in patients and rats by using two models for calculating the radiation dose: the standard MIRD schema and an ICRP model. The calculated radiation doses in 5 patients with prostate cancer who were injected firstly with diagnostic administrations 177.6 MBq (4.8 mCi) and 185 MBq (5.0 mCi) were (5.2 ± 1.2) cGy/37 MBq (or cGy/mCi) for kidneys, (3.6 ± 1.1) cGy/37 MBq for bladder wall, (3.5 ± 0.7) cGy/37 MBq for red marrow, (3.2 ± 0.5) cGy/37 MBq for normal skeleton, (0.14 ± 0.03) cGy/37 MBq for testes, and (0.37 ± 0.06) cGy/37 MBq for the whole body. 

In another study by Palmedo et al. [29] and Biersack [30], the analgesia and antitumor effects of repetitive administrations of Re-188 HEDP were evaluated in patients with metastatic prostate cancer, whereas minor toxicity problems were reported.

### 2.3. Therapeutic Ratios of Re-188 HEDP in Skeletal Metastases Treatment

According to the patient-specific 3D internal dosimetry approach by Lyra et al. [4, 31], gamma-camera images (whole-body scintigrams and SPECT) of radiopharmaceutical distribution, in patients injected with Re-188-HEDP, were analyzed to measure activity in specifically selected normal and metastatic regions of interest.Planar data (anterior and posterior images) were acquired and used for an estimation of the dose in the lesions of interest, according to the MIRD schema. For the evaluation of the spots volume estimation, cylindrical and spherical phantom of various known volumes were used. Dose rate calculations are performed for each lesion volume by using the MIRDOSE 3.1 code. A sensitive and well-established method of bone uptake quantification is the measurement of whole-body retention at 24 h after injection. But since soft tissue retention of diphosphonates is known to be as high as 30% of whole-body retention, it seems appropriate to measure soft tissue retention and net bone uptake. Calculations of the volume of spots and the corresponding absorbed dose per activity, in normal bone tissue and in skeletal metastases, were used to obtain metastatic/normal bone absorbed dose ratio (M/N ratio) and bone/red marrow mean absorbed dose ratio (B/RM ratio), in order to indicate the importance of this technique in the palliative therapy by factors. M/N ratio provides valuable information in assessing tumor control probability, normal tissue toxicity, and radiopharmaceuticals qualification and superiority, whereas B/RM ratio displays the red marrow toxicity induced by the radiopharmaceutical, a key issue for the success of the radiopharmaceuticals therapeutic use. The Normal bone absorbed dose per activity (mGy/MBq) per volume unit (N/V) and the same of metastatic lesions (M/V) were also determined. The results are presented in Table 2. 

 Breast cancer remains the major cause of cancer death in women in the developed world. Novel therapeutic modalities are needed for patients with tumors resistant to conventional therapies such as chemotherapy, hormonal treatment, and external radiation. Most of the mammal tumors express the sodium iodised symporter (NIS) enhancing the possibilities of breast cancer treatment. NIS mediates iodide accumulation and organification in the thyroid gland and thus allows the use of radioisotopes for the treatment of thyroid cancer. It has been pointed out [32] that organification of iodine is unlikely to occur in nonthyroid tumors (e.g., breast cancer cells) without transfection of NIS. However, the therapeutic gains using I-131 are minimal, due to the long physical half time (8 days), the low-energy emitted-particles, and the relatively small penetration depth up to 5 mm. A different isotope with shorter half life and better physical properties would be more adequate for therapeutic purposes, especially in the case of tumors larger than 5 mm. As an alternative option to I-131, ^99m^Tc has a proper half life (6 h), and it is trapped in a similar way to iodine but cannot be organified in the thyroid gland. Rhenium is a chemical analogue of technetium and exhibits practically identical chemical and biodistribution properties. Dadachova et al. [33] investigate the utility of Re-188 in treatment of NIS-expressing mammary tumors in a mouse model and report the results of comparative I-131 and Re-188 biodistribution in a mouse breast cancer model. Dosimetry calculations performed by extrapolation of biodistribution data to humans in the same study showed that Re-188 perrhenate is able to deliver 4.5 times higher dose to the tumor than I-131.

## 3. Conclusions

Re-188 can be produced by a W-188/Re-188 on-site generator in a convenient and inexpensive way in the majority of hospitals. Due to its favorable characteristics, Re-188 labeled radiopharmaceuticals are appealing for bone metastases. Some toxicity issues (thrombocytopenia and leukopenia) are unavoidable but manageable. Significant pain palliation is achieved especially after repeated therapy of bone metastases using Re-188 HEDP. Patient-specific 3D dosimetry allows for an accurate dose determination, and adequately high dose delivery in metastatic bone lesions to bone marrow can be achieved. Utilization of SPECT radionuclide distribution in defined ROIs can provide, through voxel slices, accurate foci volume, and the dose rate calculations are performed for each lesion volume. In this context, the concept of therapeutic ratios as described in the dosimetry section can effectively represent the results of treatment. Usage of Re-188 radiopharmaceuticals could also enhance therapeutic efficacy to other malignancies (e.g., nonresepctable liver cancer, nonmelanoma skin cancer, and breast cancer) as well as treatment of arthritis and inhibition of arterial restenosis. Therapeutic ratios in these cases could provide a powerful and reliable tool for the estimation of treatment benefits.

## Figures and Tables

**Table 1:** Physical properties of Re-188.

Property	Re-188
Physical half life	17.00 h
Maximum beta energy (abundance)	2120.4 keV (71.1%)
	1965.4 keV (25.6%)
Gamma energy (abundance)	155.0 keV (15%)
Maximum penetration in tissue	10 mm (average 3.1 mm)

**Table 2:** Re-188 HEDP therapeutic ratios.

Re-188 HEDP ratios	N/V	M/V	M/N	B/RM
Mean value	1.8	9.0	5.0	1.9

N/V and M/V are given in mGy/MBq/Voxel [4].
